# Analysis of Targeted Supplemental-Waveband Lighting to Increase Yield and Quality of Lettuce Grown Indoors

**DOI:** 10.3390/plants14071141

**Published:** 2025-04-06

**Authors:** Nathan Kelly, Erik S. Runkle

**Affiliations:** Department of Horticulture, Michigan State University, 1066 Bogue St., East Lansing, MI 48824, USA; kelly.nathan123@gmail.com

**Keywords:** anthocyanins, indoor farming, leaf coloration, light-emitting diodes, photon spectrum, photon flux density

## Abstract

Lighting from light-emitting diodes (LEDs) is one of the largest capital and operational expenses for indoor farms. While broad-waveband white LEDs are relatively inexpensive, their efficacy is lower than most narrow-band LEDs. This study aimed to determine how supplementing warm-white light with additional blue (400–499 nm), green (500–599 nm), red (600–699 nm), or far-red (700–750 nm) light influences lettuce (*Lactuca sativa*) growth and quality, and whether these effects are consistent across two photon flux densities (PFDs). We grew lettuce ‘Rouxai’ and ‘Rex’ under 90 or 180 µmol∙m^−2^∙s^−1^ of warm-white light supplemented with 40 or 80 µmol∙m^−2^∙s^−1^ of blue, green, red, far-red, or warm-white light. Supplemental far-red light increased biomass without reducing secondary metabolites. Supplemental red, far-red, and warm-white light maximized biomass, whereas additional blue light enhanced secondary metabolite concentrations and leaf coloration. Increasing the PFD increased biomass and phenolic content in ‘Rouxai’. Notably, spectral effects were consistent across PFD levels, suggesting that higher PFDs do not diminish spectral responses. These results demonstrate the potential of enriching white light to increase yield or quality in controlled-environment agriculture and provide insights for cost-effective commercial production.

## 1. Introduction

In indoor farms, electric lighting can be used to precisely control the light environment to optimize plant growth. The ability to control the photon spectrum and photon flux density (PFD) allows growers to manipulate the yield, morphology, leaf coloration, and nutritional quality of leafy green vegetables, microgreens, and other crops suitable for indoor farming. Increasing the PFD of photosynthetically active radiation (PAR; 400–700 nm) typically increases plant growth and leaf coloration, but also increases capital and operational costs [1]. In particular, increasing the percentage of red (R; 600–699 nm) light in the photon spectrum can increase biomass accumulation compared to additional blue (B; 400–499 nm) light, but at the expense of nutritional quality [2]. Furthermore, light that induces shade-avoidance responses, particularly green (G; 500–599 nm) and especially far-red (FR; 700–750 nm) light, can increase leaf expansion and sequent plant growth [3,4].

As a general rule, a 1% increase in PAR leads to a 0.75% to 1% increase in the growth and yield of horticultural crops [5]. However, the growth rate increase with the photosynthetic PFD (PPFD; 400–700 nm; µmol∙m^−2^∙s^−1^) is not linear. Electron transport rate (ETR) and the quantum yield of photosystem II photochemistry (ΦPSII) are both indicators of overall plant photosynthesis [6,7]. Lettuce (*Lactuca sativa*) ETR and ΦPSII increased when the PFD increased from 100 to 200 µmol∙m^−2^∙s^−1^ at an air temperature of 20/16 °C (day/night) and CO_2_ concentration of 400 µmol∙mol^−1^, but then began to increase at a decreasing rate as the PFD increased to 400, 600, or 800 µmol∙m^−2^∙s^−1^ [6]. However, in the same study, lettuce fresh mass (FM) increased as the PFD increased up to 600 µmol∙m^−2^∙s^−1^, despite having lower instantaneous photosynthetic rates.

Another way to characterize the light intensity for plant growth is yield photon flux density (YPFD), which is based on the relative quantum efficiency curve developed by McCree [8,9]. Unlike PPFD, which counts all photons between 400 and 700 nm equally, YPFD weights wavelengths based on instantaneous photosynthesis. Therefore, YPFD can provide a more biologically relevant measure of light quantity and considers photons beyond the PAR waveband. However, YPFD may still underestimate the contribution of far-red light to plant growth, since it can synergistically enhance photosynthesis when combined with red-light—a phenomenon described as the Emerson Enhancement Effect [10,11]. In addition to spectral efficiency, photon penetration also varies with wavelength. Green light is typically less effective than red or blue light at lower PFDs, but at higher PFDs, it penetrates deeper into leaves and dense plant canopies, increasing whole-plant photosynthesis [12]. Spectral adjustments that increase YPFD can improve photosynthetic efficiency and yield without increasing the overall PFD. In addition to biomass accumulation, increasing the PFD can also increase green leaf coloration [13] and secondary metabolite concentrations including phenolic compounds [1,14].

Light-emitting diode (LED) fixtures are widely used in commercial indoor farms to control the PFD and photon spectrum by utilizing diodes that emit narrow wavebands, broad wavebands (i.e., white light), or both [15,16,17]. Although white LEDs are less electrically effective than R or B LEDs, they are often included in lighting devices because of their low cost, broad spectrum, and high color-rendering index [15,16]. A base spectrum of white light can be enriched with one or more specific wavebands (supplemental light; SL) to achieve specific growth responses or enhance specific quality attributes. For example, adding 50 µmol∙m^−2^∙s^−1^ of B light to a warm-white (WW) photon spectrum during the entire production period increased the total phenolic concentration (TPC) and total anthocyanin concentration (TAC) of baby-leaf lettuce ‘Rouxai’ at harvest, but did not affect FM [18]. Similarly, adding 30 or 60 µmol∙m^−2^∙s^−1^ of B light to a WW + R photon spectrum for the last six days of production increased the TPC, TAC, and leaf redness of lettuce ‘Rouxai’, whereas additional G + R light at the same PFD did not [19]. FR light is not included in the PAR waveband but can increase FM by increasing photosynthesis, leaf expansion, and light capture [20,21,22,23]. However, an increased percentage of FR light in the photon spectrum decreased secondary metabolite concentration and leaf coloration [4,24]. Finally, different LED types (including those of the same waveband but with different peak wavelengths and manufacturing processes) can have different efficacies, so the selection of a photon spectrum necessitates consideration of growth responses and the costs to purchase and operate the lighting fixtures [15,16].

Modification of the PFD and/or photon spectrum during indoor plant production can regulate growth and morphology, but some greenhouse studies suggest that the SL spectrum has less effect when the solar daily light integral (DLI) is high. For example, providing various ornamental seedlings with SL in a greenhouse increased seedling dry mass (DM), height, and leaf number, but the SL spectrum had little or no effect on these metrics [25]. Similarly, pre-harvest SL provided to greenhouse-grown lettuce plants increased leaf thickness, leaf greenness, and phytochemical concentrations, but there were few effects of the photon spectrum on plant growth and quality, such as shoot FM and total phenolic content due to the high solar DLI [26]. Therefore, SL can increase crop growth and quality, but its spectrum could have little effect when added to a broad-waveband spectrum and/or moderate to high DLI. It is less clear whether these trends apply to leafy greens produced indoors under sole-source lighting. This study aimed to address this knowledge gap by evaluating the effects of supplemental narrow-band light on lettuce grown under two different background WW PFDs. Specifically, this study was designed to determine (1) how different PFDs of supplemental narrow-waveband light affect growth, phytochemical concentrations, and leaf coloration when applied to a white-light background and (2) if the same percentage of SL applied to white light at different PFDs elicits similar responses. We hypothesized the following: (1) the effects of the photon spectrum will be attenuated by a high PFD; (2) supplemental B light will increase phenolic and anthocyanin concentrations more than supplementing with other wavebands, but will also decrease leaf expansion and biomass accumulation; and (3) supplemental FR light will decrease phytochemical concentrations but increase leaf expansion and biomass accumulation. Our results indicate that supplementing white light with specific wavebands can increase the growth or quality attributes (but not both) of lettuce, and responses to the photon spectrum are similar at a low or moderate PFD. This research highlights practical applications such as increasing yield and crop traits while considering the efficacy of a photon spectrum.

## 2. Results

### 2.1. Fresh Mass and Dry Mass

**Figure 1:**
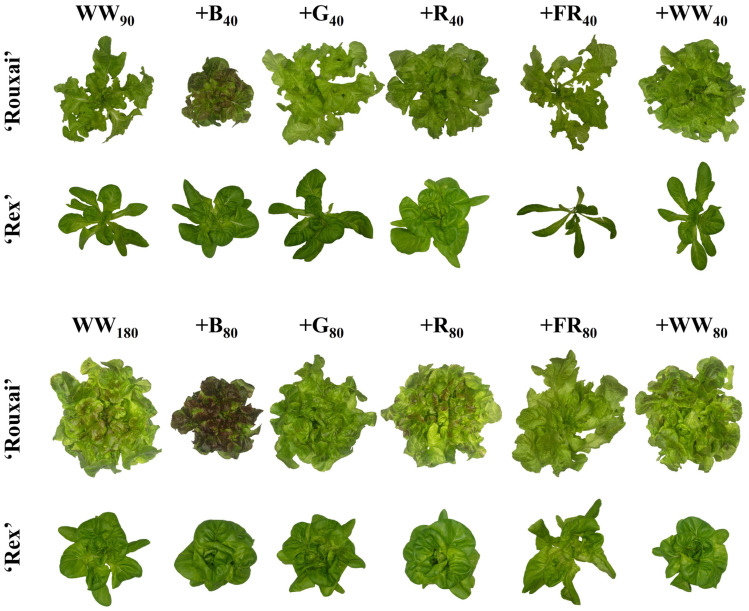
Representative plants of lettuce ‘Rex’ and ‘Rouxai’ from each light treatment. Plants were grown under a warm-white (WW) photon flux density of 90 (top two rows) or 180 µmol∙m^−2^∙s^−1^ (bottom two rows) supplemented with blue (B; 400–499 nm), green (G; 500–599 nm), red (R; 600–699 nm), far-red (FR; 700–750 nm), or WW light. Subscript values indicate the photon flux density of each waveband, in µmol∙m^−2^∙s^−1^.

**Figure 2:**
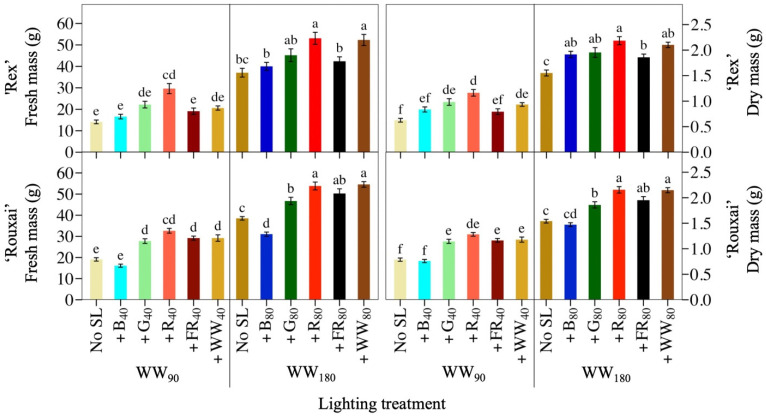
Mean shoot fresh mass and dry mass of lettuce ‘Rex’ and ‘Rouxai’ grown under a warm-white (WW) photon flux density of 90 or 180 µmol∙m^−2^∙s^−1^ plus supplemental blue (B; 400–499 nm), green (G; 500–599 nm), red (R; 600–699 nm), far-red (FR; 700–750 nm), or WW light. Subscript values indicate the photon flux density of each waveband, in µmol∙m^−2^∙s^−1^. Each bar represents the mean of two replications with ten samples per treatment and replication. Means with different letters are significantly different based on Tukey’s honestly significant difference test (α = 0.05). Error bars indicate the standard error of each treatment.

Figure 1 contains photos of representative plants of lettuce ‘Rouxai’ and ‘Rex’ from each light treatment. The WW PFD and SL waveband had a significant effect on lettuce ‘Rouxai’ and ‘Rex’ FM and DM (Figure 2; Table 1. Adding 40 µmol∙m^−2^∙s^−1^ of B light (+B_40_) to 90 µmol∙m^−2^∙s^−1^ of WW light (WW_90_) did not affect the FM of either cultivar. In contrast, 40 µmol∙m^−2^∙s^−1^ of additional G (+G_40_), R (+R_40_), FR (+FR_40_), or WW (+WW_40_) light increased ‘Rouxai’ FM, compared to no SL, by up to 71%, while only +R_40_ increased ‘Rex’ FM. At a WW PFD of 180 µmol∙m^−2^∙s^−1^ (WW_180_), the addition of 80 µmol∙m^−2^∙s^−1^ of B (+B_80_) light decreased ‘Rouxai’ FM by 19% but had no statistical effect on ‘Rex’ FM. Similar to WW_90_, lettuce ‘Rouxai’ grown with an additional 80 µmol∙m^−2^∙s^−1^ of G (+G_80_), R (+R_80_), FR (+FR_80_), or WW (+WW_80_) light had up to 42% greater FM than those grown without SL. Only the addition of R and WW light increased ‘Rex’ FM (by 41%). Doubling the WW PFD and SL PFD increased the FM of ‘Rouxai’ by 62 to 93% and ‘Rex’ by 79 to 154%. Not surprisingly, all plants grown under a total photon flux density (TPFD; 300–750 nm) of 260 µmol∙m^−2^∙s^−1^ had a greater FM than plants grown under treatments with a TPFD of 130 µmol∙m^−2^∙s^−1^, with the exception of ‘Rouxai’ grown under WW_180_B_80_.

In general, SL with the same waveband but at two PFDs had a somewhat similar effect on lettuce FM, although, on a percentage basis, SL increased growth more at the low WW PFD than the higher one. For example, lettuce ‘Rouxai’ that was grown under WW_90_R_40_ or WW_180_R_80_ had 71% and 40% more FM, respectively, than the WW treatments without SL. In addition, as the YPFD of the photon spectrum increased, so did the FM of both cultivars, except under SL B light. For example, WW_180_B_80_ had a similar YPFD to WW_180_G_80_ and WW_260_, but lettuce plants had up to 43% less FM. Additionally, lettuce ‘Rouxai’ grown under WW_180_FR_80_ had 62% more FM than plants grown under WW_180_B_80_, despite the YPFD being less.

The lettuce DM of both cultivars followed similar trends to FM (Figure 2; Table 1). At WW_90_, adding G, R, FR, or WW light increased ‘Rouxai’ DM by up to 63%, but only additional G and R light statistically increased ‘Rex’ DM. B light added to either cultivar had no effect on DM. At WW_180_, all SL wavebands increased ‘Rouxai’ and ‘Rex’ DM except +B_80_ for ‘Rouxai’. Similar to FM, SL at a low or high PFD had similar effects on FM, although the magnitude of the increase was usually greater under WW_90_ than WW_180_. For instance, adding the same percentage of G light to the low or high WW PFD increased the DM of ‘Rex’ by 57% and 26%, respectively.

### 2.2. Plant Morphology and Leaf Number

**Figure 3:**
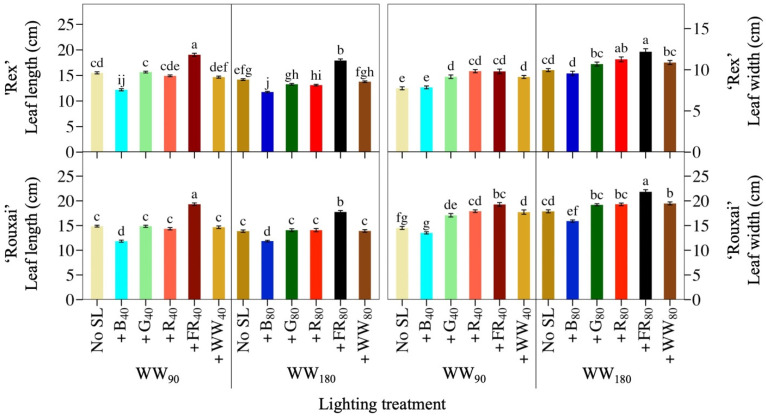
Mean leaf length and leaf width of the fifth fully expanded leaf of lettuce ‘Rex’ and ‘Rouxai’ grown under warm-white (WW) light without or with supplemental light (SL). See Figure 1 for treatment information. Subscript values indicate the photon flux density of each waveband, in µmol∙m^−2^∙s^−1^. Each bar represents the mean of two replications with ten biological samples per treatment and replication. Means with different letters are significantly different based on Tukey’s honestly significant difference test (α = 0.05). Error bars indicate the standard error of each treatment.

The photon spectrum and the TPFD influenced plant and leaf morphology (Figure 3; Table 1; Table 2). The addition of 40 or 80 µmol∙m^−2^∙s^−1^ of B light to WW_90_ or WW_180_ decreased the leaf length of lettuce ‘Rouxai’ and ‘Rex’ by up to 21%. In contrast, at both WW PFDs, adding 40 or 80 µmol∙m^−2^∙s^−1^ of FR light increased the leaf length of both cultivars by 23% to 30%. None of the other SL wavebands had an effect. Adding G, R, FR, or WW light to WW_90_ similarly increased the leaf width of ‘Rex’, by up to 27%, but additional B light to ‘Rex’ had no effect. There were similar spectral effect trends on the leaf width of lettuce ‘Rouxai’, except that FR SL increased leaf width slightly more than additional WW light. At WW_180_, only +R_80_ and +FR_80_ increased ‘Rex’ leaf width; the other SL treatments had no effect. ‘Rouxai’ leaf width, on the other hand, decreased by 11% when +B_80_ light was added to WW_180_ and increased by 22% and 9% under the +FR_80_ or +WW_80_ treatments. Similar to leaf length, the plant diameter of both cultivars increased when FR light was added to both WW PFDs and decreased under additional B light (Table 3). At WW_90_, +FR_40_ increased the plant diameter of ‘Rouxai’ to a greater extent than the same percentage of FR light applied at twice the WW PFD. Finally, adding 40 or 80 µmol∙m^−2^∙s^−1^ of WW light to the low or high WW PFD did not influence ‘Rouxai’ plant diameter, but at WW_180_, +WW_80_ decreased ‘Rex’ plant diameter.

The number of leaves greater than 2 cm of both cultivars was primarily affected by the TPFD, although supplemental light did influence leaf number as well. Lettuce grown under the higher TPFD had more leaves than those grown under the lower TPFD. For example, lettuce grown under WW_180_ had three to four more leaves than lettuce grown under WW_90_. Additionally, lettuce grown under a higher WW PFD plus FR (WW_180_FR_80_) had fewer leaves than plants grown under WW_180_ without FR SL (Table 2). In contrast, adding G or R light to WW_90_ increased the leaf number of both cultivars by up to 26%. At WW_180_, the addition of SL did not increase the lettuce leaf number of either cultivar except for R_80_ added to ‘Rouxai’, which increased the leaf number by 12%.

### 2.3. Total Phenolic and Anthocyanin Concentrations

**Figure 4:**
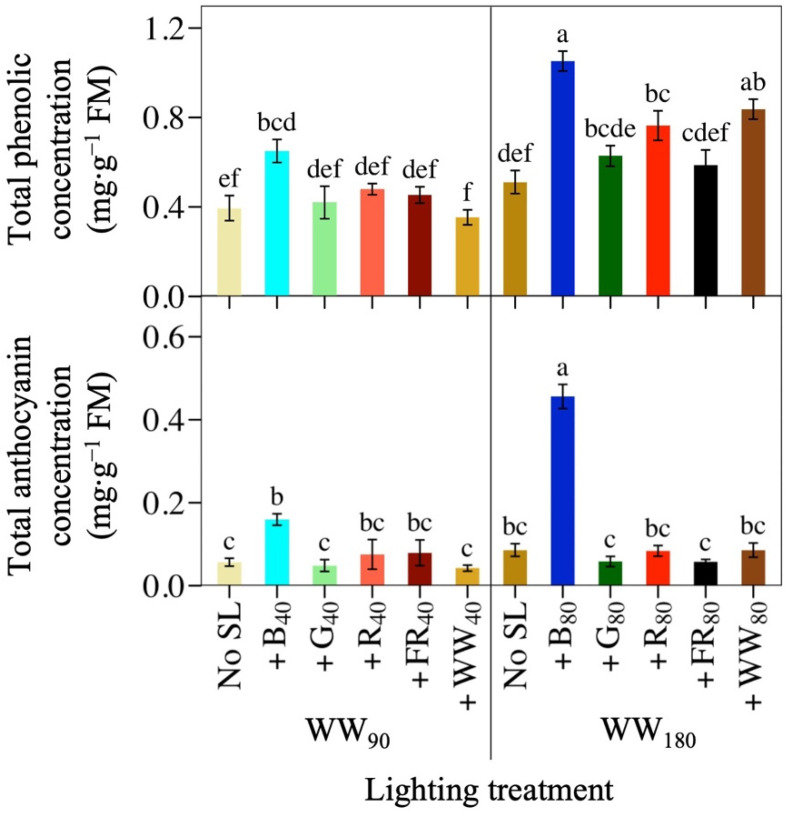
Mean total phenolic concentration and total anthocyanin concentration of lettuce ‘Rouxai’ grown under warm-white (WW) light without or with supplemental light (SL). See Figure 1 for additional treatment information. Subscript values indicate the photon flux density of each waveband, in µmol∙m^−2^∙s^−1^. Each bar represents the mean of two replications with three biological samples per treatment and replication. Means with different letters are significantly different based on Tukey’s honestly significant difference test (α = 0.05). Error bars indicate the standard error of each treatment.

Supplemental B light was the most effective waveband at increasing both the TPC and TAC of lettuce ‘Rouxai’, while doubling the PFD of any photon spectrum had less of an effect (Figure 4; Table 1). At WW_90_, +B_40_ increased TPC and TAC by 65% and 182%, respectively. No other supplemental waveband influenced TPC or TAC, although plants under +R_40_ and +FR_40_ had a similar TAC to +B_40_ and the WW_90_ control. At WW_180_, +B_80_ increased lettuce TPC and TAC by 105% and 430%, respectively, compared to no SL. Additionally, ‘Rouxai’ grown under the higher TPFD had greater TPC and TAC than under the low TPFD. For example, lettuce grown under WW_180_B_80_ had 106% and 423% greater TPC and TAC, respectively, than plants grown under the same photon spectrum but half the TPFD. At the high TPFD, +R_80_ light and +WW_80_ increased TPC, but the same SL wavebands had no effect on TPC at WW_90_.

### 2.4. Leaf Coloration and SPAD

**Figure 5:**
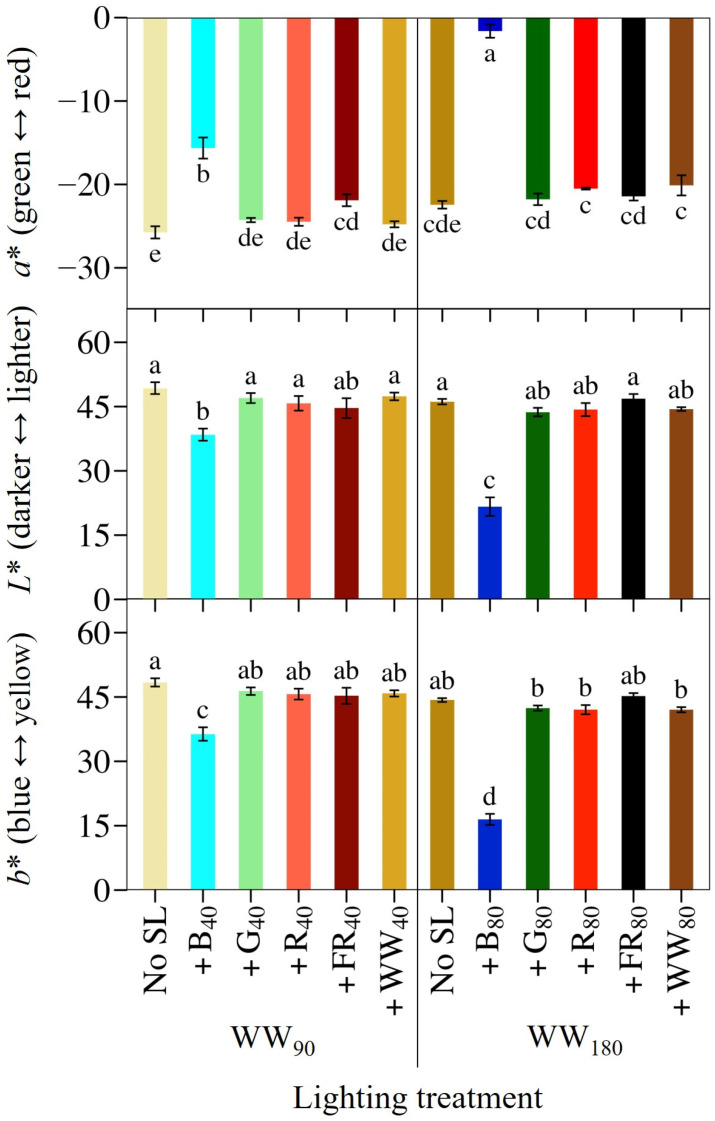
Mean leaf pigmentation indicated by L*a*b* coloration index values of lettuce ‘Rouxai’ grown under warm-white (WW) light without or with supplemental light. See Figure 1 for additional treatment information. Subscript values indicate the photon flux density of each waveband, in µmol∙m^−2^∙s^−1^. Each bar represents the mean of two replications with three biological samples per treatment and replication. Means with different letters are significantly different based on Tukey’s honestly significant difference test (α = 0.05). Error bars indicate the standard error of each treatment.

We quantified ‘Rouxai’ leaf coloration using the L*a*b* color space. At WW_90_, +B_40_ increased leaf redness (more positive a* value) by 39% and +FR_40_ increased leaf redness by 15% (Figure 5). Furthermore, +B_40_ slightly increased the blueness (lower b* value) and darkness (lower L* value) of lettuce leaves. At WW_180_, +B_80_ was the only treatment that increased leaf redness, blueness, and darkness; they were 93% redder than those grown without SL and 90% redder than plants grown under the same photon spectrum but at a TPFD of 130 µmol∙m^−2^∙s^−1^.

Doubling the WW PFD without SL increased the SPAD of lettuce ‘Rouxai’ and ‘Rex’ by 34% and 20%, respectively (Table 2). Additional B light increased the SPAD of both cultivars regardless of the WW PFD, while FR light decreased the SPAD of both cultivars only when added to WW_180_.

## 3. Discussion

### 3.1. Phytochrome-Mediated Responses to Red and Far-Red Light

Phytochrome (phy) is the primary plant photoreceptor that senses R and FR light to initiate signal cascades leading to morphological and growth adaptations [27]. The increase in leaf expansion of lettuce grown under supplemental FR light can be attributed to an increase in the FR fraction of the photon spectrum [27,28,29]. The additional FR light increases the FR fraction and decreases the phytochrome photoequilibrium (PPE), which is the ratio of the biologically active form of phytochrome (Pfr) relative to the total phytochrome pool, which is comprised of Pfr plus the inactive form of phytochrome (Pr) [8]. The internal PPE (iPPE) considers the spectral distortion that occurs as light travels through the leaf [30]. The iPPEs of our various treatments were somewhat similar to each other (0.66–0.77) when the photon spectrum consisted of only WW light or WW light plus supplemental B, G, or R light (Table 3). When FR light was added to the photon spectrum, the iPPE decreased to 0.31, indicating a strong shift in phytochrome forms. As FR light constitutes more of the photon spectrum, phyB is converted to the Pr form, decreasing the PPE or iPPE. As the phyB pool is converted to more of the Pr form of phyB, it dissociates from phytochrome-interacting factor (PIF) 4 and PIF5, permitting them to move to and accumulate in the nucleus to promote the expression of shade-avoidance genes [28,31,32,33]. Furthermore, PIFs interact with the CONSTITUTIVELY PHOTOMORPHOGENIC 1 (COP1) and SUPRESSOR OF PHYA (SPA) enzyme complexes, which target ubiquitinate, and degrade phyB as well as photomorphogenesis transcription factors like ELONGATED HYPOCOTYL 5 (HY5) that are involved in stem and leaf elongation [34,35,36,37]. Removing phyB from the nucleus, either by dissociation from PIF4 and PIF5 or degradation mediated by COP1/SPA, allows for the increased expression of shade-avoidance genes that increase cell elongation and subsequently leaf surface area.

An increase in leaf expansion leads to greater light capture, which can increase whole-plant photosynthesis and sequent growth. Therefore, an increase in leaf area or canopy diameter often correlates with an increase in biomass accumulation [3,21,29,38]. However, the addition of FR light to a white spectrum led to plants with fewer but larger leaves. For example, the addition of FR light to an R + B spectrum increased individual leaf area, but lettuce plants had fewer leaves, leading to a similar total leaf area among lighting treatments [39]. FR light can also directly stimulate photosynthesis, especially when paired with R light, by preferentially stimulating photosystem I [10,11,40]. Thus, incorporating FR light into a photon spectrum that contains R light increases plant leaf expansion and growth both indirectly and directly [41]. Furthermore, supplemental B light inhibited FM accumulation of lettuce ‘Rouxai’, but only at the higher TPFD (+B_80_), while the extension growth (leaf length) of both cultivars was inhibited at both TPFDs. Adding R or WW light to the photon spectrum also increased the FM of both cultivars by increasing PAR with a higher quantum yield than B light [9], while supplemental B light (+B_80_) inhibited the biomass accumulation of lettuce ‘Rouxai’ despite having a similar YPFD to light treatments containing supplemental R, G, or WW light.

In the current study, increasing the FR PFD (+FR_40_ or +FR_80_) increased the leaf length and width of both cultivars, regardless of the TPFD. The FM of lettuce ‘Rouxai’ grown under additional FR light also increased relative to no SL, but was similar to plants grown under only WW light at an increased TPFD or under WW + R light. In contrast, the FM of ‘Rex’ was similar regardless of whether FR light was included in the photon spectrum. Other studies have also reported that FR SL increased leaf expansion and growth. For example, lettuce grown under 200 µmol∙m^−2^∙s^−1^ of R + B light plus 50 µmol∙m^−2^∙s^−1^ of FR light delivered during the day or at the end of the day increased leaf area by nearly 50% [42]. The FR SL also increased FM, especially when delivered during the entire day. In another study, adding ≈160 µmol∙m^−2^∙s^−1^ of FR to a white-light background increased leaf length, width, and stem length, as well as FM and DM [24]. Additionally, when the TPFD remained constant but the FR light percentage increased, lettuce FM increased and was correlated with an increase in leaf area [4,29]. Therefore, FR light, either when added or substituted for another waveband, increases leaf expansion, light interception, and subsequent growth.

### 3.2. Supplemental Light or the PFD Increases Phenolic Compounds and Leaf Coloration

Phenolic compounds are a broad group of secondary metabolites including flavonoids and specific color-causing antioxidants such as anthocyanins. Increasing the B PFD greatly increased both the TPC in leafy greens and anthocyanins [19,24,43,44]. Cryptochrome 1 (cry1) is the primary photoreceptor that controls phenolic compound biosynthesis in response to high-energy, short-waveband light such as B [45]. *CHALCONE SYNTHASE (CHS)* is the gene that encodes the enzyme involved in the first committed step of the flavonoid biosynthesis pathway, and is expressed under ultraviolet or B light in plants with a functional cry1 [46,47]. Its expression is associated with increased concentrations of phenolic compounds [48].

Increasing the PFD of ultraviolet, B, or total light generally increases phytochemical concentrations [19,26,43]. In this study, at the higher PFD (WW_180_), +R_80_ and +WW_80_ light increased lettuce ‘Rouxai’ TPC but not TAC. In another study, the TPC of two lettuce cultivars was not modified by R SL, but concentrations of individual phenolic compounds like chicoric acid, rutin, and kaempferol were greater in lettuce ‘New Red Fire’ than with white light without SL or with B SL [49]. In contrast, increasing the percentage of R light in a broad-waveband spectrum increased lettuce TPC [24]. Increasing the PFD from 400 to 700 µmol∙m^−2^∙s^−1^ at ambient CO_2_ concentration increased the TPC of both red- and green-leaf lettuce [14]. Additionally, high light (800 µmol∙m^−2^∙s^−1^) for at least one day increased lettuce TPC [50]. Therefore, lettuce TPC and some specific phenolic compounds are sensitive to specific wavebands of light, such as B light, and concentrations can also be increased by increasing the PFD of broad-waveband light. At least in lettuce and when applied at the same PFD, the results here and those of Kelly and Runkle [19,43] indicate that B light is the most effective waveband at increasing TPC and anthocyanins, which are important for red-leaf lettuce pigment accumulation.

### 3.3. Interactions Between Supplemental Light and the PFD

The specific waveband of SL and the WW PFD interacted to influence various plant traits such as ‘Rouxai’ FM, TPC, and TAC, and ‘Rex’ plant diameter and leaf length. For example, ‘Rouxai’ FM decreased when supplemental B light was applied, but only at the higher PFD. Additionally, +R and +WW light increased ‘Rouxai’ TPC only at the higher TPFD, while TAC increased under +B light, but to a greater extent under the higher TPFD. Few studies have investigated the effects of narrow-waveband SL applied to broad-waveband light at different TPFDs with the same spectral distribution. In this study, SL similarly affected a given plant trait at both WW PFDs, but the magnitude depended on the WW PFD even though the SL percentage remained the same. For example, +B light increased leaf redness, but to a greater extent at the higher TPFD. Furthermore, FR light increased leaf length more when added to the lower WW PFD than twice the FR PFD at twice the WW PFD. Further research should investigate if these trends persist at even higher TPFDs (e.g., 500–800 µmol∙m^−2^∙s^−1^).

Increasing the PFD by increasing the intensity of the same photon spectrum (additional WW light) and incorporating SL of a specific waveband both increase electrical costs, but is one more effective at increasing biomass accumulation or improving plant quality than the other? Our study indicates that doubling the WW PFD from 90 to 180 µmol∙m^−2^∙s^−1^ was more effective at promoting biomass accumulation than adding any SL waveband to 90 µmol∙m^−2^∙s^−1^ of WW light. There were exceptions with leaf expansion in which supplemental FR light applied to a low WW PFD increased leaf length more than doubling the WW PFD, although it did not lead to an increase in FM. When considering supplementing a white light with narrow-waveband light, it is important to consider the efficacy of the LEDs. For example, if supplemental G or FR light added to a white background similarly increases FM, FR LEDs may be preferred because of their higher photon efficacies [15]. In our study, supplemental R light and an additional 40 or 80 µmol∙m^−2^∙s^−1^ of WW light led to the greatest biomass accumulation. Assuming R LEDs have an average photon efficacy of 3.6 µmol∙J^−1^ and WW LEDs have a photon efficacy of about 2.7 µmol∙J^−1^, supplemental R light increased lettuce FM more per unit of energy input.

## 4. Materials and Methods

### 4.1. Plant Material and Growth Conditions

We sowed red oakleaf lettuce ‘Rouxai’ and green butterhead lettuce ‘Rex’ (Johnny’s Selected Seeds, Winslow, ME, USA) seeds in a temperature-controlled growth room (the Controlled Environment Lighting Laboratory) at Michigan State University. We sowed the seeds in 200-cell (2.5 cm × 2.5 cm) rockwool plugs (AO 25/40 Starter Plugs; Grodan, Milton, ON, Canada) that were presoaked in deionized water with a pH of 4.5 that was adjusted using 10% sulfuric acid (H_2_SO_4_). After seed sow, the lettuce seeds germinated at 23 °C under a 24 h·d^–1^ photoperiod and a TPFD of 180 µmol∙m^−2^∙s^−1^ from warm-white (peak = 639 nm, correlated color temperature = 2700 K) LEDs. On day 3, we shortened the photoperiod to 20 h·d^–1^ for the rest of the production cycle. To increase humidity during germination and early seedling growth, we covered the seedling trays with clear plastic domes from day 0 to 5. From day 0 until day 8, before the seedlings were transplanted, we hand-irrigated the seedlings with deionized water supplemented with magnesium sulfate (Epsom salt; Pennington Seed, Inc., Madison, GA) and a water-soluble fertilizer (12N–4P_2_O_5_–16K_2_O RO Hydro FeED; JR Peters, Inc., Allentown, PA, USA) with the following nutrients (in mg∙L^–1^): 125 N, 42 P, 167 K, 73 Ca, 49 Mg, 39 S, 1.7 Fe, 0.52 Mn, 0.56 Zn, 0.13 B, 0.47 Cu, and 0.13 Mo. The pH and electrical conductivity (EC) were monitored daily using a pH/EC meter (HI9814; Hanna Instruments, Woonsocket, RI, USA) and were adjusted as needed to maintain a pH of 5.6 and an EC of 1.6 mS∙cm^−1^.

The growth room consisted of four vertical hydroponic growing racks that had three canopies on each rack with recirculating nutrient solutions. On day 8, we randomly separated the seedlings and transplanted 30 uniform plants into each of 12 floating 36-cell rafts (Beaver Plastics, Ltd., Acheson, AB, Canada) with 2.5 cm wide holes spaced 20 × 15 cm apart. The three outermost cells at the ends of each raft were left empty to minimize edge effects and ensure uniform lighting among plant samples. Plants were provided the same nutrient solution as previously described but at a 20% higher concentration (i.e., 150 mg N∙L^–1^). We measured the nutrient solution daily and adjusted it using additional fertilizer, potassium bicarbonate, and H_2_SO_4_ to maintain a pH and EC of 5.7 and 1.9 mS∙cm^−1^, respectively. The air temperature was a constant 23 °C. We measured and calculated the mean (±SD) canopy temperature (Rep. 1 = 24.8 ± 0.9 °C; Rep. 2 = 25.0 ± 0.8 °C), relative humidity (Rep. 1 = 47.7 ± 5.6%; Rep. 2 = 45.2 ± 9.0%), and CO_2_ concentration (Rep. 1 and 2 = 411 ± 21 µmol∙mol^−1^) during each replication. Additional information about equipment, experimental conditions, and sensors can be found in detail in Kelly et al. [13].

### 4.2. Light Treatments

**Figure 6:**
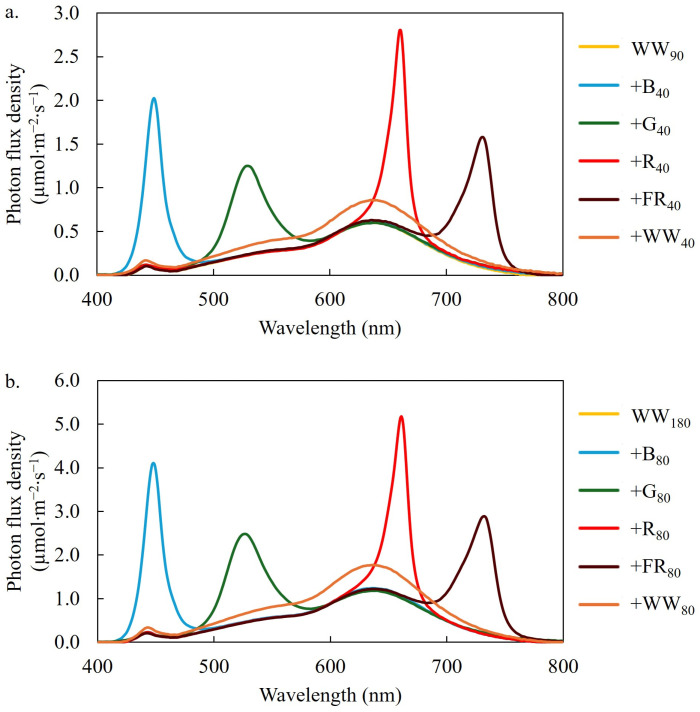
Photon spectrum of the 12 lighting treatments. The treatments consisted of a warm-white (WW) photon flux density of 90 (**a**) or 180 (**b**) µmol∙m^−2^∙s^−1^ supplemented with 40 (**a**) or 80 (**b**) µmol∙m^−2^∙s^−1^ of blue (B; 400–499 nm), green (G; 500–599 nm), red (R; 600–699 nm), far-red (FR; 700–750 nm) or WW light. Subscript values indicate the photon flux density of each waveband, in µmol∙m^−2^∙s^−1^.

We delivered 12 lighting treatments from day 8 until the plants were harvested on day 28 (Table 3, Figure 6). Each lighting treatment consisted of a base spectrum of WW light at a TPFD of 90 (low) or 180 µmol∙m^−2^∙s^−1^ (high). We added 40 or 80 µmol∙m^−2^∙s^−1^ of narrow-band SL using B (peak = 449 nm), G (peak = 526 nm), R (peak = 664 nm), or FR (peak = 733 nm) LEDs to the low or high TPFDs, respectively. We also created two treatments that consisted of an additional 40 or 80 µmol∙m^−2^∙s^−1^ of WW light. This enabled the comparison of narrow-band SL on lettuce growth and quality attributes at the same percentage of the TPFD at a low (130 µmol∙m^−2^∙s^−1^) and high (260 µmol∙m^−2^∙s^−1^) TPFD. We measured the TPFD and photon spectrum of each treatment at nine locations at the plant canopy level using a spectroradiometer (PS200; Apogee Instruments, Inc., Logan, UT, USA). The mean PFDs of each waveband range are reported in Table 3.

### 4.3. Experimental Design and Replication

This experiment was arranged as a randomized complete block design with two independent replications in time. For each replication, each light treatment had ten biological samples per lettuce cultivar for growth and morphology measurements. For biochemical assays and coloration analysis, three randomly selected plants per treatment and replication were used. Three technical replicates were created for each biochemical assay. Randomization was performed by reassigning treatment positions among the racks in the growth room to negate any localized environmental effects.

### 4.4. Biochemical Analyses and Data Collection

Immediately after harvest (day 28), we collected fresh leaf tissue of ‘Rouxai’ to measure TPC and TAC. To measure TPC, we collected 0.5 g of direct light-exposed leaf tissue from three separate biological samples, immediately froze them in liquid nitrogen, and then stored them in a −80 °C freezer until analysis. We analyzed the samples using a spectrophotometer according to the protocol used in Kelly and Runkle [19], which was developed with slight modifications from the Ainsworth and Gillespie [51] protocol. TAC analysis was conducted similarly, except we collected 0.3 g of plant tissue and analyzed the samples using a modified version of the Lee et al. [52] pH differential method (AOAC Official Method 2005.2) previously described in Kelly and Runkle [19].

On day 28, before destructive plant measurements, we measured the relative chlorophyll concentration of ten randomly selected ‘Rouxai’ and ‘Rex’ plants using a SPAD meter (SPAD-502; Konica Minolta Sensing, Inc., Osaka, Japan) by measuring and averaging three spots on one fully expanded leaf exposed to direct light. Additionally, for the red-leaf cultivar ‘Rouxai’ we took overhead pictures of three randomly selected plants to measure leaf coloration using an R code developed [53] to determine the lightness (black: L* = 0; white: L* = 100), redness (green: a* = −128; red: a* = 127), and blueness (blue: b* = −128; yellow: b* = 127) of each pixel of an imported TIFF picture. The L*, a*, and b* values of each pixel were generated and averaged to quantify the average coloration of an entire plant from overhead.

After harvest, we randomly selected ten plants from each treatment and cultivar for morphological data collection. These plants were not used for biochemical analysis. We cut and harvested all plant tissue and weighed each using an analytical balance (AG245; Mettler Toledo, Columbus, OH). Additionally, we measured the plant diameter (cm), leaf length (cm) and width (cm) of the fifth fully expanded leaf, and counted the number of leaves longer than 2 cm. Finally, we packed all plant tissue into paper bags and dried them in a drying oven (Blue M, Blue Island, IL) for 7 days at 60 °C before weighing them with the same balance.

### 4.5. Statistical Anaylsis

We performed statistical analysis using R statistical analysis software [54] using the R package’s ‘dplyr’ [55] and ‘agricolae’ [56]. Two-way analysis of variance (ANOVA) tested the main effects of supplemental wavebands, the WW PFD, and their interaction. Post hoc comparisons were performed using Tukey’s honestly significant difference test (α = 0.5).

## 5. Conclusions

Both the photon spectrum and flux density regulate lettuce growth, morphology, and coloration. It is important to identify the specific plant traits desired when supplementing white light with narrow-waveband light, or whether increasing the PFD of the same spectrum is sufficient to elicit those attributes, which would alleviate the need to incorporate additional LED types into a lighting system. In this study, doubling the TPFD of WW light had the most pronounced effect on FM, DM, and leaf number, while leaf expansion (plant diameter and leaf length) increased with supplemental FR light. Although supplementing WW light with FR light increased leaf expansion, additional R light was the most effective at increasing FM. Furthermore, B light increased TPC, TAC, and leaf coloration, but suppressed leaf expansion and FM compared to WW light with additional R or WW light. Finally, one should consider the efficacies and costs of individual LED types as well as electricity costs to increase the PFD to incrementally increase biomass accumulation.

## Figures and Tables

**Table 1:** Results of two-factor analysis of variance for lettuce ‘Rouxai’ and ‘Rex’. The *p* values indicate the main effects of the supplemental light (SL) waveband, warm-white (WW) photon flux density (PFD), or their interaction on lettuce growth, morphology, relative chlorophyll concentration (SPAD), total phenolic concentration (TPC), total anthocyanin concentration (TAC), and leaf coloration (L*a*b*). nd = not determined.

Factor	‘Rouxai’			‘Rex’		
	SL Waveband	WW PFD	SL Waveband × WW PFD	SL Waveband	WW PFD	SL Waveband × WW PFD
Fresh mass	<0.001	<0.001	0.009	<0.001	<0.001	0.163
Dry mass	<0.001	<0.001	0.048	<0.001	<0.001	0.627
Plant diameter	<0.001	0.005	<0.001	<0.001	<0.001	<0.001
Leaf length	<0.001	<0.001	0.027	<0.001	<0.001	<0.001
Leaf width	<0.001	<0.001	0.363	<0.001	<0.001	0.376
Leaf number	<0.001	<0.001	0.064	<0.001	<0.001	0.081
SPAD	<0.001	<0.001	0.062	<0.001	<0.001	<0.001
TPC	<0.001	<0.001	0.008	nd	nd	nd
TAC	<0.001	<0.001	<0.001	nd	nd	nd
L*	<0.001	<0.001	<0.001	nd	nd	nd
a*	<0.001	<0.001	<0.001	nd	nd	nd
b*	<0.001	<0.001	<0.001	nd	nd	nd

**Table 2:** Mean plant diameter, leaf number, and relative chlorophyll concentration (SPAD) of lettuce ‘Rouxai’ and ‘Rex’ grown under a warm-white (WW) photon flux density (PFD) of 90 or 180 µmol∙m^−2^∙s^−1^ without or with supplemental blue (B; 400–499 nm), green (G; 500–599 nm), red (R; 600–699 nm), far-red (FR; 700–750 nm), or WW light. Each value represents the mean of two replications with ten biological samples per treatment and replication. Means with different letters are significantly different based on Tukey’s honestly significant difference test (α = 0.05).

Cultivar	WW PFD	Treatment	Plant Diameter (cm)	Leaf Number	SPAD
Rouxai	90	WW_90_	24.2 c	11.3 e	12.8 d
		+B_40_	20.1 d	10.7 e	16.1 c
		+G_40_	25.6 c	13.1 d	13.5 d
		+R_40_	24.7 c	13.8 cd	15.5 c
		+FR_40_	33.0 a	11.4 e	12.4 d
		+WW_40_	25.0 c	13.0 d	13.8 d
	180	WW_180_	24.2 c	14.8 bc	17.1 bc
		+B_80_	21.4 d	13.2 d	19.2 a
		+G_80_	24.3 c	15.2 ab	17.9 ab
		+R_80_	24.5 c	16.1 a	18.5 ab
		+FR_80_	29.3 b	13.3 d	15.6 c
		+WW_80_	24.5 c	16.4 a	18.4 ab
Rex	90	WW_90_	30.2 b	13.5 ef	17.5 fg
		+B_40_	23.4 g	14.4 de	21.6 cd
		+G_40_	30.0 b	15.5 cd	18.0 f
		+R_40_	28.4 bc	16.9 bc	20.4 de
		+FR_40_	35.3 a	12.2 f	16.0 g
		+WW_40_	28.7 bc	15.0 de	18.6 ef
	180	WW_180_	27.2 cd	18.1 ab	21.0 d
		+B_80_	21.4 fg	17.9 ab	25.1 a
		+G_80_	24.9 ef	19.0 a	20.0 de
		+R_80_	24.8 ef	19.2 a	23.1 bc
		+FR_80_	33.7 a	15.7 cd	17.7 fg
		+WW_80_	25.5 de	19.5 a	24.2 ab

**Table 3:** Total photon flux densities (300–750 nm), yield photon flux densities, and individual waveband photon flux densities for each lighting treatment. Treatments consisted of warm-white (WW) light delivered at 90 (WW_90_) or 180 (WW_180_) µmol∙m^−2^∙s^−1^ plus 40 or 80 µmol∙m^−2^∙s^−1^ of blue (B, 400–499 nm), green (G, 500–599 nm), red (R, 600–699 nm), or far-red (FR, 700–750 nm) light. Subscripted values denote individual photon flux densities in µmol∙m^−2^∙s^−1^.

	Photon Flux Density (µmol∙m^−2^∙s^−1^)					
Treatment	Total	Yield	Blue	Green	Red	Far-Red
WW_90_	87.1	74.1	5.7	26.9	48.4	6.1
+B_40_	131.4	106.6	48.2	27.6	48.9	6.8
+G_40_	132.8	109.3	8.3	68.1	48.8	7.4
+R_40_	134.4	116.8	6.9	26.8	93.1	7.6
+FR_40_	137.3	88.1	5.7	28.0	54.5	49.2
+WW_40_	128.3	107.7	9.7	38.8	69.3	10.3
WW_180_	179.4	152.4	12.0	55.5	99.1	12.8
+B_80_	267.2	217.4	98.3	56.1	99.7	13.0
+G_80_	265.5	218.0	17.4	137.8	95.9	14.2
+R_80_	264.3	230.4	11.9	54.9	183.4	13.0
+FR_80_	269.4	172.5	11.9	54.5	106.5	96.6
+WW_80_	258.6	219.2	17.9	79.5	142.2	19.0

## Data Availability

Data will be made available upon request.

## Abbreviations

The following abbreviations are used in this manuscript:

B	Blue
DLI	Daily light integral
DM	Dry mass
EC	Electrical conductivity
ETR	Electron transport rate
FM	Fresh mass
FR	Far-red
G	Green
iPPE	Internal phytochrome photoequilibrium
LED	Light-emitting diode
PAR	Photosynthetic active radiation
PFD	Photon flux density
PIF	Phytochrome interaction factor
PPE	Phytochrome photoequilibrium
R	Red
SL	Supplemental light
TAC	Total anthocyanin concentration
TPFD	Total photon flux density
TPC	Total phenolic concentration
WW	Warm-white
YPFD	Yield photon flux density
ΦPSII	Quantum yield of photosystem II photochemistry
